# Relationship between adiponectin and insulin resistance among a cohort of obese adolescents and young adults in a tertiary institution

**DOI:** 10.4314/gmj.v59i4.4

**Published:** 2025-12

**Authors:** Okechukwu O Ezekpo, Segun A Atolani, Ejemhen A Ekhaiyeme, Olasoji I Ibidapo, Morenike B Kolapo, Cecilia K Okunlola, Chidiebere V Ugwueze

**Affiliations:** 1 Department of Medicine, Afe Babalola University, Ado-Ekiti (ABUAD) & ABUAD Multi-System Hospital, Ado-Ekiti, Ekiti State, Nigeria; 2 Department of Medicine, Afe Babalola University, Ado-Ekiti & Federal Teaching Hospital Ido-Ekiti, Ekiti State, Nigeria; 3 Department of Internal Medicine, Lagos State University Teaching Hospital, Ikeja, Lagos, Nigeria; 4 Department of Medicine, Ekiti State University Teaching Hospital, Ado-Ekiti, Ekiti State, Nigeria; 5 Department of Medicine, Alex-Ekwueme Federal University, Ndufu Alike Ikwo, Abakaliki, Ebonyi State, Nigeria

**Keywords:** Serum Adiponectin, Obesity, Insulin resistance, Adolescents and Young adults

## Abstract

**Objectives:**

To demonstrate the relationship between adiponectin and insulin resistance among adolescents and young adults

**Design:**

A cross-sectional descriptive study.

**Setting:**

This study was conducted within a tertiary institution and the medical outpatient department (MOPD) of its adjourning tertiary health-care institution.

**Participants:**

The study was conducted among students of a tertiary institution and patients attending the ABUAD Multi-system Hospital, aged 16-24 years.

**Main outcome measure:**

The mean levels of serum adiponectin in obese adolescents and young adults and its relationship with clinical correlates of insulin resistance

**Results:**

Mean serum adiponectin level was 6.8 µg/ml, which is comparable to what has been obtained in other studies. Statistically non-significant negative correlations were noted between serum adiponectin and the homeostasis model assessment of insulin resistance (HOMA-IR), as well as with the clinical correlates of insulin resistance such as Body Mass Index and diastolic blood pressure.

**Conclusion:**

This study demonstrated no significant correlation between serum adiponectin and markers of insulin resistance among these participants and may not be a useful marker of insulin resistance among this group of individuals; hence, its routine measurement may not be appropriate. A follow-up study involving more obese subjects may, however, be more revealing.

**Funding:**

None declared

## Introduction

The World Health Organisation (WHO) defines overweight and obesity as abnormal or excessive accumulation of fat that may impair health.^1^ There has been a steady increase in the prevalence of obesity since the early 1980s, with its attendant complications, and this has posed significant health challenges among the population globally.^2^

It is a growing healthcare concern worldwide and is an endemic health problem in both developed and developing countries.^3^ It is associated with many chronic diseases, including hypertension, dyslipidaemia, type 2 diabetes mellitus and some cancers.^4^ Obesity is a chronic and stigmatising disease that is causally related to serious medical illnesses, impaired quality of life, and considerable economic burden due to increased health care costs and loss of productivity.^5^

About 12 million people in Nigeria were estimated to be obese in 2020, with prevalence considerably higher among women. Nutritional and epidemiological transitions driven by demographic changes, rising income, urbanisation, unhealthy lifestyles and consumption of highly processed diets appear to be driving an obesity epidemic in the country.^6^

Adolescent obesity is a public health problem that affects not only physical growth and development, but also has an impact on social and emotional issues and experiences in stigmatising situations.^7^ They are often victims of these stigmatisations and their obesity results in a wide range of unsolicited psychosocial and medical costs, including impacting adult physique, poor self-esteem and self-image, problems of integration with peers, depression, anxiety and the chronic diseases attributable to excess adiposity.^8^,^9^

It has also been established that the pre-diabetes rate in overweight adolescents is 2-6 times higher than that of normal weight adolescents.^10^ In some countries like the US, close to 1 in 5 adolescents and 1 in 4 young adults are living with prediabetes, the prevalence of which is higher among obese individuals.^11^ Generally, young adults are often presumed healthy and are therefore rarely the subjects of obesity research (as opposed to children and the elderly).^12^ Overweight/obesity in adolescents and young adults, if left unchecked, will constitute future risks for associated non-communicable diseases such as diabetes and cardiovascular disease.^8^,^13^,^14^

There is an important relationship between Obesity and Insulin resistance. Suraamornkul et al.^15^ reported that obesity is associated with increased insulin resistance. Akande et al.^16^, in a study conducted among the Nigerian population, confirmed a positive correlation between overweight/obesity and insulin resistance.

Obesity associated chronic low-grade inflammation is a major cause of decreased insulin sensitivity, which makes obesity a major risk factor for insulin resistance.^17^-^19^ Obesity may increase the expression of certain inflammatory cytokines and activate multiple signalling pathways, both of which contribute to the pathogenesis of insulin resistance by interfering with insulin signalling and action.^20^

Functionally, adiponectin (a 244-amino-acid-long collagen-like hormone protein, also referred to as AdipoQ) modulates energy metabolism, including glucose and fatty acid oxidation, insulin sensitivity, inflammation, and atherosclerosis, and is the most abundant circulating adipokine secreted by adipose tissue.^21^

It has also been demonstrated that adiponectin, as an anti-inflammatory marker, inversely correlates with indices of obesity such as waist circumference, BMI and Waist-hipratio (WHR), while pro-inflammatory markers such as hsCRP and IL-6 showed positive correlation with BMI.^22^ Several studies,^23^,^24^ have shown the relevance of serum adiponectin as an anti-inflammatory marker which is usually reduced in obesity, T2DM and other conditions associated with insulin resistance.

This study evaluated the usefulness of adiponectin as a marker of insulin resistance in obese Nigerian adolescents and young adults attending a Tertiary Institution.

## Methods

The study was conducted among students of Afe Babalola University of Ado-Ekiti (ABUAD) and patients attending the ABUAD Multi-system Hospital, aged 16-24 years. A simple random sampling method was used in this cross-sectional descriptive study.

Subjects who were known or suspected to have chronic debilitating diseases such as chronic heart failure, chronic liver disease, chronic renal failure, subjects with diabetes mellitus or those who were on long-term steroid therapy, diuretics, beta-blockers, as well as subjects who had acute febrile illness in the preceding week before the study and subjects presenting with diabetic emergencies were all excluded.

Ethical approval was sought for and granted by the University and hospital's Ethics and Research Committee - Research and Ethics Committee, Afe Babalola University, Ado-Ekiti (ABUAD), Nigeria. Reference number: AMSH/REC/OE/046. Written informed consent was also obtained from participants.

The clinical history and relevant examination were obtained and documented. Anthropometry and blood pressure were measured and documented using standard protocols.^25^,^26^

Laboratory assessment for all subjects included the collection of 5-7 mL of venous blood sample at once under aseptic procedure from the cubital fossa of each subject between 8.00 and 8.30 a.m. after an overnight fast of at least 8 hours but not more than 12 hours. All subjects were assessed for insulin resistance using the homeostatic model assessment of insulin resistance (HOMA-IR), as described by Matthews et al.^27^,^28^. The formula proposed by Matthews *et al* is:

HOMA-IR = (fasting plasma insulin (µU/ml) × fasting plasma glucose (mmol/l))/22.5

The constant is a normalising factor, the product of fasting plasma insulin of 5µU/mL and plasma glucose of 4.5mmol/L obtained from an “ideal” and “normal” individual. Therefore, for an individual with normal insulin sensitivity, HOMA-IR = 1.

The IR cut-off point in blacks approximates the mean value of 1.95 + 1 standard deviation (SD) (0.1) obtained in Ghanaians with normal FBG and blood pressure using the HOMA model^29^ Serum adiponectin level was measured using enzyme-linked immunosorbent assay (Elab-science, USA).

### Data Analysis/Calculations

The data collected for the study were analysed using the Statistical Package for Social Sciences (SPSS) version 23.0 for Windows (IBM Corp., Armonk, N.Y., USA). Descriptive statistics, such as means and standard deviations, were calculated for the continuous variables (anthropometric parameters, fasting blood glucose, fasting plasma insulin, fasting plasma adiponectin, lipid profiles), while frequencies and percentages were computed for the categorical variables (participants' age and obesity grades). The Pearson correlation technique (Relationship between serum Adiponectin levels and clinical and biochemical correlates of Insulin resistance), independent-samples t-tests, and the F-test (Analysis of variance) were employed for inferential statistics. P-value < 0.05 was considered statistically significant.

## Results

A total of 90 participants were involved in the study. The respondents' ages ranged from 16 to 24 years, with a mean age of 19.4 ± 1.7 years. More than half (52.2%) of the respondents belonged to a common age group of 16-19 years, while others (47.8%) were in the age range of 20 to 24 years. Approximately one-third of the study participants were male (32.2%), and the remaining two-thirds (67.8%) were female.

A total of 37.8% of the study participants had a positive recent history of hyperphagia, while almost all the participants (98.9%) admitted to the regular consumption (≥ 3 per week) of calorie-dense foods. The majority of the study participants (63.3%) had a family history of obesity, specifically in their mothers, while 68.9% had a positive family history of obesity in other First-Degree Relatives (FDR). Almost half of the study participants (47.8%) had a positive history of hyperphagia in childhood, while a larger percentage (63.3%) were noted to have had a history of obesity in childhood. (Figure 1) Most participants (87.8%) were not engaged in regular exercise, and more than half (51.1%) reported a history of sedentary behaviour.

Table 1 shows that the mean weight of study participants was 102.7kg, and the mean height was 1.69 m. The mean body mass index (BMI) of the study participants was 35.7kg/m^2^, with a minimum of 30.0kg/m2 and a maximum of 49.3kg/m2. The mean values for waist circumference and hip circumference were 103.1 cm and 123.7cm, respectively. The mean waist–hip ratio was 0.83, while the mean systolic and diastolic blood pressures were 126mmHg and 84mmHg, respectively.

**Table 1:** Clinical examination findings of Study participants

	Mean	SEM	Std. Dev.	Minimum	Maximum
**Weight (kg)**	102.79	1.78	16.89	74.0	148.0
**Height (metres)**	1.69	0.01	0.09	1.53	1.91
**Body mass Index (kg/*m*^2^)**	35.75	0.52	4.91	30.0	49.3
**Waist circumference (cm)**	103.1	1.33	12.64	77.0	141.0
**Hip circumference (cm)**	123.79	0.99	9.39	104.0	156.0
**Waist-Hip-ratio (WHR)**	0.83	0.009	0.09	0.66	1.14
**Systolic Blood Pressure**	126.37	1.46	13.82	90.0	156.0
**Diastolic Blood Pressure**	84.41	1.25	11.86	53.0	106.0

SEM: standard error of mean; Std. Dev.: standard deviation

More than half (54%) of the study participants had grade 1 obesity, while 28% had grade 2 obesity, and morbid obesity was noted in 18% of the subjects. The majority of the study participants (87%) were obese by both Body mass index and waist circumference measurements, while 13% were observed to be obese with only their BMI.

Table 2 shows that the mean fasting blood glucose was 4.86 mmol/L among the study participants. Mean fasting plasma insulin levels were 2.95 µIU/ml, while the mean fasting adiponectin levels were 6.8µg/ml. The mean HOMA-IR was noted to be 0.64

**Table 2:** Laboratory results of study participants

	Mean	SEM	Std. Dev.	Minimum	Maximum
**Fasting Blood Glucose**	4.86	0.11	1.08	3.57	13.65
**Fasting Plasma Insulin Level**	2.95	0.1	0.9	1.69	7.22
**Fasting Plasma Adiponectin Level**	6.82	0.46	4.4	1.61	22.46
**High-Density Lipoprotein - Cholesterol**	1.4	0.03	0.33	0.76	2.8
**Triglyceride**	1.09	0.12	1.12	0.38	9.81
**Low-Density Lipoprotein - Cholesterol**	2.62	0.08	0.75	0.80	6.14
**Total Cholesterol**	4.54	0.09	0.90	3.00	8.59
**HOMA – IR**	0.64	0.03	0.25	0.29	2.02

SEM: standard error of mean; Std. Dev.: standard deviation

The mean serum adiponectin levels were significantly higher in females than in males (p=0.032). Similarly, serum adiponectin was higher in the younger age group (16-19) than in the older age group (20-24); however, this difference was not statistically significant, and there was no significant association between serum adiponectin and obesity class. (Table 3)

**Table 3:** Mean levels of serum adiponectin by sex, age and body mass index

	Mean	SD	SEM	Minimum	Maximum	p-value
**Gender**						
**Male**	6.14	3.37	0.43	1.61	18.72	0.03*
**Female**	8.25	5.84	1.08	1.64	22.49	
**Total**	6.82	4.4	0.46	1.61	22.49	
**Age**						
**16-19**	7.4	5.09	0.74	1.64	22.49	0.19
**20-24**	6.18	3.44	0.52	1.61	14.47	
**Total**	6.82	4.4	0.46	1.61	22.49	
**BMI**						
**Grade 1**	6.86	4.04	0.58	1.64	22.3	0.798
**Grade 2**	7.13	5.68	1.14	1.61	22.49	
**Morbid**	6.19	3.21	0.8	1.66	12.11	
**Total**	6.82	4.4	0.46	1.61	22.49	

*: *P*-value <0.05

The linear relationship between serum adiponectin and clinical correlates of insulin resistance, such as WC, WHR, BMI, SBP, and DBP, was assessed using Pearson's Correlation in Table 4.

**Table 4:** Relationship between serum adiponectin levels and clinical and biochemical correlates of Insulin resistance

	*R*	p-value
**Body mass Index (kg/m^2^)**	-0.06	0.596
**Waist circumference (cm)**	0.08	0.449
**Hip circumference (cm)**	0.10	0.34
**Waist-Hip-ratio (WHR)**	0.10	0.354
**Systolic Blood Pressure**	0.11	0.294
**Diastolic Blood Pressure**	-0.03	0.761
**HOMA – IR**	-0.19	0.075

*r*: Pearson Correlation coefficient; *: *P*-value <0.05

There was a negative correlation between serum adiponectin and body mass index, diastolic blood pressure, and HOMA-IR; however, this was not statistically significant. In contrast, there was a non-significant positive correlation between serum adiponectin and waist circumference, hip circumference, waist-to-hip ratio, and systolic blood pressure.

## Discussion

Serum adiponectin levels have been associated with correlates of insulin resistance, such as HOMA-IR, waist circumference, BMI, and lipid profile, among obese adolescents and young adults. Some studies have shown that this adipocytokine, with its anti-inflammatory and anti-atherogenic properties, may be a useful biomarker of obesity and predictive index of insulin resistance in these patients.^30^ Some other studies done in Africans, however, did not establish such a relationship.^31^ This study comprised 90 obese adolescents and young adults.

In this study, obesity was determined using both Body mass index (BMI) and waist circumference. The BMI is widely recognised as a useful and effective tool for the determination and subclassification of obesity among adolescents and young adults.^32^ In addition to the BMI, the National Institute of Health (NIH) also recognises waist circumference as a tool by which abdominal obesity can be measured, as this is noted to correlate better with insulin resistance.^33^

The age range of subjects in this study was from 16 to 24 years, and more than half of the study population (52.2%) were in the 16-19 age bracket, while 47.8% of participants were in the 20-24 years age bracket, with the mean age of the study population being 19.4years ± 1.7.

It is worth noting that since the participants are from a single institution and include both students and patients, they may not accurately represent the general adolescent or young adult population in Nigeria.

Almost all the study participants (98.9%) had a positive history of regular consumption (≥ 3 per week) of calorie–dense foods such as pastries, egg yolk, red meat, pizza, fizzy drinks, etc., and about 37.8% of the participants also had a recent history of eating excessively. Some studies have related the lifestyle of youths to risk factors for obesity, including excess energy consumption as well as excessive eating.^34^,^35^

The greatest obesogenic environmental risk is diet; the high caloric, overly processed, and poor quality of certain diets have increased genetic and epigenetic obesity risk and rendered people overfed and undernourished.^36^ Family history of obesity in the mother or other first-degree relatives was found in 63.3% and 68.9% of study participants, respectively. 63.3% of participants also had a history of obesity in childhood. The risk of becoming an obese adult is increased both by having been obese as a child and by having at least one obese parent. The risk of adult obesity rises with increasing age and with the severity of obesity in childhood. For example, the risk of being obese at 21 to 29 years of age ranged from 8% for persons who were obese at 1 to 2 years of age and had non-obese parents to 79% for persons who were obese at 10 to 14 years of age and had at least one obese parent. Although persons who were obese at 1 to 2 years of age and had lean parents did not have an increased risk of obesity in adulthood, persons who became obese after 6 years of age had a greater than 50% chance of becoming obese adults.^37^

The mean serum adiponectin level in the study participants was 6.82 µg/ml, with a minimum of 1.61 µg/ml and a maximum of 22.46 µg/ml. In a study done among Indian adolescents by Jain et al in 2017.^38^ Similar mean values of serum adiponectin (6.0 µg/ml) were observed. Nri Ezedi et al, however, found a lower mean serum adiponectin in their study among prepubertal Nigerians; the lower level of adiponectin obtained in their study may not be unconnected with the younger age of their study population compared with our study^39^

The mean serum adiponectin was significantly higher among the female subjects (8.25µg/ml), than their male counterparts (6.14µg/ml) (p=0.032). This is attributable to differences in body adiposity between the sexes, and various studies have shown that sex hormones also affect the production rate of adiponectin.^40^,^41^

Serum adiponectin levels by age group were comparable, with subjects aged 16–19 years having a mean adiponectin level of 7.4 µg/ml, whereas those aged 20–24 years had a mean of 6.18 µg/ml. The age gap between these groups apparently did not make much of a difference, though it has been noted that circulating levels of Adiponectin are influenced by age.^42^,^43^

According to BMI, the mean serum adiponectin levels for grade 1 obese subjects were 6.86 µg/ml, whereas those for subjects with grades 2 and 3 obesity were 7.13 µg/ml and 6.82 µg/ml, respectively. These differences in adiponectin values, however, were not statistically significant. In various studies done, BMI has been found to correlate with adiponectin levels in young adults.^44^,^45^

The HOMA-IR is a surrogate marker of insulin resistance and was used in this study to assess the relationship between serum adiponectin and insulin resistance. Waist circumference (WC), Body mass Index (BMI), waist–to–hip ratio (WHR), systolic and diastolic blood pressure, are not only clinical, anthropometric, and laboratory parameters but also markers of insulin resistance and were used in this study as clinical and biochemical correlates of insulin resistance. In this study, Serum adiponectin levels correlated negatively with Body mass index (r =-0.06, p = 0.596) and HOMA-IR (r = -0.19, p = 0.075), though not statistically significant and positively with waist circumference (r = 0.08, p = 0.449), hip circumference (r = 0.10, p = 0.34), waist-hip ratio (r = 0.10, p = 0.354), and Systolic blood pressure (r = 0.11, p = 0.294),; however, these were also not statistically significant. The above were all weakly positive and negative correlations. Similar findings from certain studies have demonstrated a relationship between adiponectin and Body mass index, as well as HOMA-IR and waist circumference as markers of obesity and insulin resistance.

In a study by Varda et al. 30 on obese Slovenian adolescents, adiponectin showed a strong, statistically significant negative correlation with BMI across all three observed groups (p < 0.001). It was therefore concluded that adiponectin and other obesity-related biological markers tested (ghrelin & leptin) are useful early markers for identifying patient groups at cardiovascular risk.

A similar observation was made by Khabour et al^44^ in a study done among obese female Jordanian young adults, where it was demonstrated that adiponectin and BMI correlated negatively. However, further comparisons showed BMI or adiponectin were not related to G276T and I164T Single Nucleotide Polymorphisms (SNP) of the ADIPOQ gene in these young adult women.

These are contrary to findings by Hassan et al^31^ who studied Egyptian obese adolescents and observed a poor correlation between adiponectin and BMI, HOMA-IR, and WC and therefore concluded that there was no direct link between these parameters. The authors were of the notion that what applies to other ethnic populations might not apply to the African population. Racial differences, as proposed by these authors, as well as Lee et al in 2006,^46^ who had similar findings in their study, which was between African Americans and their Caucasian peers, may be largely responsible for these variations. The above relationship between adiponectin and other variables, showing weak and non-significant correlations, underscores the need for further, larger cohort studies.

This study has some limitations. The study setting did not allow for generalisation of our findings to all other adolescents and young adults in Nigeria, given that only one institution was studied. However, a one-centre study, despite its limitations, provides a basis for future multicentre studies. The relatively small sample size and cross-sectional nature preclude adequate causal inference. However, the study's findings contribute to the limited body of research in this field in our environment.

Body mass index (BMI) was used to assess obesity in this study. While widely used to evaluate obesity, this method has limitations, including its inability to predict the degree of adiposity in patients. Assessing other biologic markers of obesity and insulin resistance, such as Leptin, Ghrelin, and Tumour necrosis factor alpha, would have enhanced the findings in this study, but this could not be done due to the huge cost that would be involved and the lack of laboratory facilities for the assay of some of the biomarkers.

## Conclusion

Serum adiponectin was not associated with HOMA-IR or other clinical correlates of insulin resistance, such as Body mass index and diastolic blood pressure (DBP). However, as expected, there was a negative correlation with these parameters. Other clinical correlates, such as waist circumference, hip circumference, waist-to-hip ratio (WHR), and systolic blood pressure (SBP), showed positive correlations but were not statistically significant. All the above exploratory findings, however, were weakly positive or negative.

Routine measurement of serum adiponectin may have limited utility as a sole marker of insulin resistance among obese adolescents and young adults in our environment. However, further studies with larger sample sizes and diverse populations are needed to validate the findings from this research.
